# Supplementary material for: MiR-23b Promotes Porcine Preadipocyte Differentiation via SESN3 and ACSL4
Source: Cells. 2022 Jul 29;11(15):2339. doi: 10.3390/cells11152339 (PMC9367261; doi:10.3390/cells11152339)
Supplement: Supplementary file 1 [file cells-11-02339-s001.zip › cells-1813134-supplementary/Supplementary files/Table S1.pdf]

Table S1 Primer sequences of mRNAs

Primer names	Primers sequences (5′→3′)	Product size/bp
SESN3	F: GAAACAATGGGCCGGGCTTA	179
	R: AGAATGGAAACCATTGGTGACG	
ACSL4	F: ACTCTGTTCAAGATAGGGTATG	189
	R: GCAGCAGAAGCAGACATTC	
PPARγ	F: AGAGTATGCCAAGAACATCC	261
	R: AGGTCGCTGTCATCTAATTC	
FABP4	F: AAGTCAAGAGCACCATAACC	119
	R: GATACATTCCACCACCAACT	
C/EBPα	F: AGCCAAGAAGTCGGTAGA	150
	R: CGGTCATTGTCACTGGTC	
C/EBPβ	F: AAGAGTAAGACCAAGAAGACC	139
	R: GCTCCAGGACCTTATGCT	
18S rRNA	F: CCCACGGAATCGAGAAAGAG	122
	R: TTGACGGAAGGGCACCA	
