# Supplementary material for: MiR-23b Promotes Porcine Preadipocyte Differentiation via SESN3 and ACSL4
Source: Cells. 2022 Jul 29;11(15):2339. doi: 10.3390/cells11152339 (PMC9367261; doi:10.3390/cells11152339)
Supplement: Supplementary file 1 [file cells-11-02339-s001.zip › cells-1813134-supplementary/Supplementary files/Table S2.pdf]

Table S2 Primer sequences of miRNAs

Primers names	Primers sequences (5′→3′)
U6	F: CTCGCTTCGGCAGCACA
	R: AACGCTTCACGAATTTGCGT
miR-23b	RT: GTCGTATCCAGTGCAGGGTCCGAGGTA TTCGCACTGGATACGACTGGTAA
	F: GCGATCACATTGCCAGGGA
	R: AGTGCAGGGTCCGAGGTATT
